# Higher Prevalence of Cancer in Patients with Diabetic Foot Syndrome

**DOI:** 10.3390/jcm13051448

**Published:** 2024-03-01

**Authors:** Chiara Goretti, Alessandro Prete, Alex Brocchi, Elisabetta Iacopi, Letizia Pieruzzi, Alberto Piaggesi

**Affiliations:** 1 Diabetic Foot Section, Department of Endocrinology and Metabolism, University of Pisa, 56126 Pisa, Italy; elisabettaiacopi@gmail.com (E.I.); pieruzziletizia@gmail.com (L.P.); alberto.piaggesi@med.unipi.it (A.P.); 2 Diabetology Unit, Department of Endocrinology and Metabolism, University of Pisa, 56126 Pisa, Italy; alessandro.prete22@gmail.com (A.P.); alexbrocchi@gmail.com (A.B.)

**Keywords:** diabetic foot syndrome, cancer, diabetes, death, comorbidities

## Abstract

**Background**: Diabetes mellitus (DM) is associated with a higher prevalence of many forms of cancer. Diabetic foot syndrome (DFS) is associated with higher risk of lower limb amputation and mortality not all explainable with a cardiovascular profile at greater risk compared with DM patients without DFS. DFS could be associated with an increasing cancer incidence. To explore a possible link between DFS and cancer, comparing two cohorts of patients (DFS+ and DFS−) with a cohort of superimposable non-DM controls. **Methods:** We retrospectively analysed the databases of our department for all consecutive patients admitted between January 2019 and December 2021, selecting all DM pts, and sorting DFS+ pts, admitted for foot complications, from DFS− ones, admitted for other reasons. Cases of pancreatic cancer as well as cancer-related admissions were excluded. Patients were compared to non-DM patients admitted for non-oncological medical problems. The primary endpoint was to compare the prevalence of cancer among the groups, while the secondary endpoint was to look for predictors for cancer in the groups studied. **Results:** A cohort of 445 consecutive DM inpatients (222 DFS+ and 223 DFS−) and 255 controls were studied. Cancer prevalence in DFS+ group was significantly higher than in DFS− (*p* = 0.008) and controls (*p* = 0.031), while no differences were observed between DFS− and the controls. Univariate regression analysis showed a significant association between cancer and DFS (*p* = 0.007), age at admission (*p* ≤ 0.001), years of diabetes (*p* = 0.017) and haemoglobin concentration [Hb] (*p* = 0.030). In the multivariate regression analysis with DFS, age at admission and [Hb], only DFS (*p* = 0.021) and age at admission (*p* ≤ 0.001) persisted as independent factors associated with cancer. **Conclusions:** A higher prevalence of cancer in DFS+ patients than in DFS− patients and non-diabetic controls is reported. DFS and age can both be considered independent predictors of cancer in patients with DM.

## 1. Introduction

The association between diabetes mellitus (DM) and cancer, two of the most relevant and severe pathologies in the world today, has been hypothesized since the 1990s when many authors demonstrated a higher prevalence of many forms of cancer (liver, breast, colon, endometrium, pancreas, kidney) in patients with DM [1].

Despite the fact that, some pathogenetic mechanisms, like hyperglicemia, hyperinsulinism, chronic inflammation and oxidative stress actually link DM to cancer, it is still uncertain if this relationship can really be considered of causative nature or rather be the consequence of common pathologic conditions shared by DM and cancer, like obesity, aging and physical inactivity. Moreover, it is not clear if some subset of diabetic patients, could be more at risk for cancer, when compared to other patients with diabetes and to general population [2,3,4,5,6].

Several epidemiological studies have appreciated the impact of duration and intensity of hyperglycaemia on the initiation and development of chronic complications of diabetes. However, even if glycaemic profiles alone could not explain per se the development and worsening of diabetic complications, we know, from some extensive randomized, controlled clinical trials focusing on the relationship glucose control/complications development, (DCCT/EDIC and UKPDS), that the quality of glycaemic control can exert a protective or detrimental impact throughout the clinical history of both in Type 1 (T1DM) and in Type 2 (T2DM) diabetes mellitus [3,4]. Chronic hyperglycaemia results in the formation of vascular, immunological, neuropathic complications. [5].

Diabetic foot syndrome (DFS) is a multi-morbid pathology encompassing both acute and chronic manifestations at both systemic and local levels, affecting diabetic patients with complications at the lower limbs; it is a chronic recurrent disease affecting 10% of diabetic population worldwide, with the highest risk of non-traumatic lower limb amputation, but also with a high risk of death, which is 2.5 times higher than in diabetic patients without DFS [1,6].

Despite the fact that it is generally assumed in the literature that the excess mortality in DM patients can be related to cardiovascular pathologies, there is still no solid evidence that other conditions, like cancer, could not contribute to this [7,8]. 

The high rate of mortality and the progressiveness of the syndrome, which is extremely prone to recur, led some authors to compare DFS to cancer, and to change the perspective according to which the disease should be framed and managed; from an acute to a chronic remitting–relapsing disease, with the outcomes not set on healing but rather on remission, which are typical oncological concepts.

This similitude, which not only encompasses the clinical profile of the two conditions, but is also related to the mortality rates, which for DFS ranges between 40% and 50% in five years, together with the already established link between DM and cancer, might allow us to hypothesize a link also between DFS and cancer [6].

In view of some of the features that characterize DFS, like immunopathy and neuropathy, but are also involved in the pathogenesis of many forms of cancer, the hypothesis of a higher oncological risk in DFS patients is sound and worth verification [8].

Our DF multidisciplinary unit in a university hospital is a referral centre for a population of 4 M, with 20 K with DM, 15 K with DFS, following more than 600 patients per year for more than 30 years, both as outpatients and during admissions, and thus is in a favourable position to explore possible comorbid association between DFS and other pathological conditions, like cancer.

We aimed to explore the hypothesis of a link between DFS and Cancer, comparing diabetic patients with DFS (DFS+) and without (DFS−) DFS with non-diabetic superimposable controls.

## 2. Patients, Materials and Methods

We retrospectively searched the databases of our department for all the consecutive patients admitted between January 2019 and December 2021 and reviewed their medical record. According to the following inclusion criteria: they should be affected by DM (ICD-code: 250.00) and, for DFS+, be admitted for complications of the lower limb (ICD-Codes: 440.24, 730.17).

**Figure 1:**
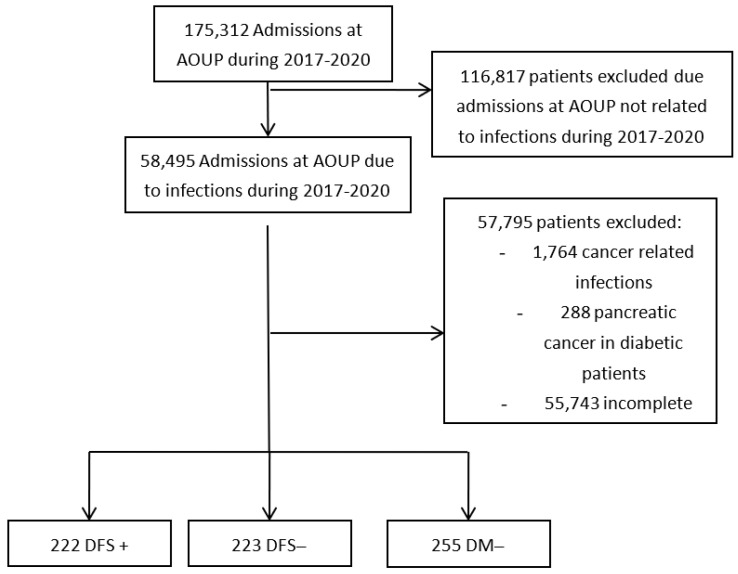
Consort diagram of the study.

Among all patients that were admitted to our hospital in that period of time, we excluded patients affected by pancreatic cancer, or cancer-related infections; from the remaining records we sorted out those admitted because of DF-related problems (DFS+), mainly infection and critical limb ischemia, from the ones admitted for other reasons (DFS−) (Figure 1).

We compared the two groups with a control group of non-diabetic patients admitted in the same period for non-oncological medical problems.

In particular, the population studied is composed of three different subgroups of patients: the first group is composed of consecutive patients admitted for DFS in the acute phase, in need of foot surgical procedures and revascularization, the second group is composed of diabetic patients without DFS, mainly admitted for metabolic decompensation, and the control group is composed of non-diabetic patients, admitted for systemic infections or for other acute systemic diseases.

We collected data directly from the medical records of the patients, focusing on diabetes, its chronic complications and on comorbidities as well as the presence or history of cancer, determined by anamnesis or clinical and instrumental findings. We also searched the clinical history for ischemic heart disease or heart failure, and vascular or carotid artery disease.

## 3. Outcomes

Our primary aim was to compare the prevalence of cancer among the groups under observation, while our secondary aims were to analyse the relationships between cancer and other possible predictors like age, sex, MACE, renal insufficiency, anaemia, inflammatory status, type of diabetes and its duration, obesity, smoke and alcohol habits.

As per the standard protocol of our hospital, patients upon admission provided formal consent for the introduction of their data to a database and to their anonymized use in an aggregate form.

Being a retrospective analysis of clinical data, according to local regulations, it was not necessary to submit it to the Ethical Committee.

## 4. Statistical Analysis

Continuous variables are expressed as means, medians and standard deviations, while categorical variables as frequencies and percentages. Data were compared using Chi-squared and Fisher’s exact tests for the categorical data and using Student’s *t*-test for the continuous variables. The statistical analysis was performed using SPSS 29.0 software (SAS Institute, Cary, CA, USA). A *p* value of less than 0.05 was considered statistically significant.

## 5. Results

Four-hundred-forty-five consecutive DM inpatients (222 DFS+ and 223 DFS−) and 255 non-DM controls were studied.

The demographic characteristics of the patients are reported in Table 1.

The proportion of males was significantly higher in the DF group than in the other ones (*p* < 0.008). DF group patients were significantly younger than DM patients (*p* < 0.001), but older than non-DM ones (*p* > 0.005).

The prevalence of type 2 diabetes (T2DM) was similar in DFS+ and DFS− (94.6% vs. 96.7%) (Table 2). Diabetes diagnosis occurred earlier in DFS+ (48.8 ± 15.6 yrs) than DFS− patients (58.2 ± 15.3 yrs), meaning a longer exposure to dis-glycaemia (median 19.0 vs. 13.0) (*p*: 0.017). Diabetes control was worse in DFS+ than DFS− patients (HbA1C 60.8 ± 19 vs. 55.3 ± 16.6 mmol/mol) (*p*: 0.009) despite the more frequent use of insulin (74.2% vs. 45.1%) and more intensive insulin regimens (basal bolus regimen 73.0% vs. 53.5%). Prescription of anti-glycaemic drugs with recognized cardiovascular protection (GLP1RAs and SGLT2i) did not differ between the two groups (21.5% vs. 15.5%) (Table 2).

**Figure 2:**
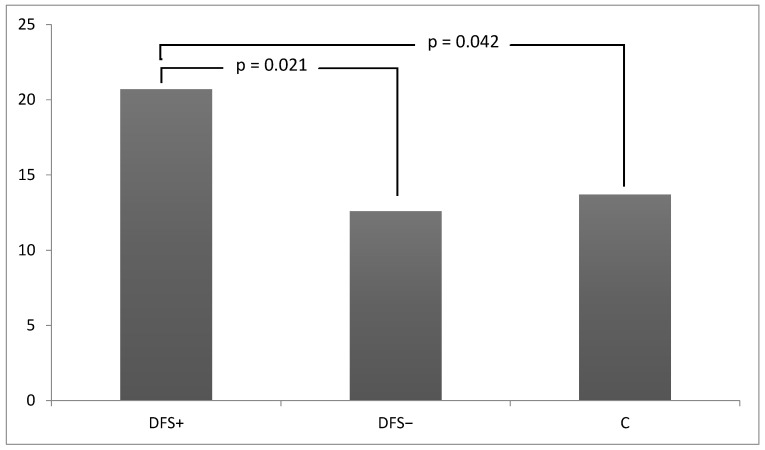
Cancer prevalence in DFS+, DFS− and Controls (C).

The overall prevalence of cancer in the whole study population was 15.3% (107/700); in DFS+ group (20.7%, 46/222) it was significantly higher compared to DFS− group (11.7%, 26/223, *p* = 0.008) and the controls (13.3%, 34/255, *p* = 0.031). While no difference emerged between the DFS− and control group for this item (*p* = 0.594) (Figure 2).

Analysing the distribution of the types of cancer among these three groups, we observed that skin (12/46, 26.1%), blood (9/46, 19.6%) and colon (8/46, 17.4%) cancers were the most common in the DFS+ group, in DFS− patients, breast (5/26, 19.2%), kidney (4/26, 15.4%) and skin (3/26, 11.5%) cancers were the most common, while in the non-DM patients, breast (8/34, 23.5%) and prostate (4/34, 11.8%) cancers were the most common (Table 3).

A history of MACE and AF was significantly less prevalent in the DFS+ group (48.6% and 15.6%) than in the DFS− group (59.4% and 26.9%; *p*: 0.027); while in the controls (22.5% and 10.6%), it was lower compared to the DFS+ group, *p* (*p* < 0.01).

DFS+ and DFS− patients showed lower levels of total Hb (11.6 ± 4.7, 11.7 ± 2.1 vs. 13.2 ± 2.1 g/dL) and higher levels of creatinine (1.25, 1.23 vs. 0.93 mg/dL, *p* < 0.001) compared to non-DM ones. C-reactive protein (CRP) levels were significantly lower in the DFS+ group than in the DFS− and control groups (2.35 vs. 10.25 mg/dL, *p* > 0.01), while procalcitonin levels and neutrophil count did not differ between the groups. On the other hand, lymphocyte count was significantly higher in the DF group than in the DM and control groups (1.68 vs. 1.01 e 1.00, *p* > 0.01).

Univariate regression analysis showed a significant association between the presence or history of cancer and DFS+ (Exp B 1.787, CI 95% 1.172–2.723, *p* = 0.007), age at admission (Exp B 1.032, CI 95% 1.015–1.050, *p* ≤ 0.001), duration of diabetes (Exp B 1.027, CI 95% 1.005–1.051, *p* = 0.017) and haemoglobin levels (Exp B 0.862, CI 95% 0.782–0.950, *p* = 0.030). At multivariate regression analysis with DF, age at admission and haemoglobin levels, DF (Exp B 1.740, CI 95% 1.088–2.784, *p* = 0.021) and age at admission (Exp B 1.037, CI 95% 1.017–1.057, *p* < 0.001) persisted as independent factors associated with presence or history of cancer (Table 4).

## 6. Discussion

Our study points out a higher prevalence of cancer in DFS+ patients, which is actually two times more frequent in this subset of diabetic patients, when compared with DFS− patients and non-diabetic controls.

Although the high prevalence of cancer in DFS+ was expected, because the already reported higher frequency of cancer in DM, the extent of these findings is nevertheless surprising and deserves some interpretation.

Diabetes and cancer are both multifactorial and heterogeneous diseases, characterized by a complex network of pathogenetic mechanisms; over time, multiple mechanisms have been described as potential causal factors in cancer pathogenesis in people affected by diabetes, like hyperglycaemia, insulin resistance and hyperinsulinism, increased bioavailability and bioactivity of IGF1, oxidative stress, chronic inflammation, adiposity, altered intestinal microbiota, alteration of sex hormones and genetic background [9,10].

However, it remains unclear whether Type 2 diabetes (T2D) is causally related to cancer or rather the association is confounded by other factors connected to both T2D and cancer. Indeed, the increasing prevalence and earlier onset of T2D coincides with that of being overweight and obesity. Therefore, it has been argued that the association between T2D and cancer could be non-causal and rather reflect a true, causal link between obesity and cancer [9,10]. Delineating the causality between T2D and specific cancers is important for identifying high-risk groups that could be efficiently targeted for early detection strategies and preventative interventions. Detecting and treating cancers at an earlier stage will thus lead to improved patient outcomes and survival.

Diabetes has been independently associated with the incidence of several types of cancer, particularly of the liver, breast, endometrium, pancreas, colon and kidney. Type 1 and type 2 diabetes are associated with an excess risk of incidence and mortality for overall and several site-specific cancers [11].

Furthermore, it has been reported that the prevalence of cancer in patients with DM varies according to tumour site, and that the association may result from shared risk factors between T2DM and cancer (older age, obesity, physical inactivity and smoking habit) [12].

DM could represent an independent risk factor for higher cancer-related mortality, particularly evident for colon, pancreas and breast cancers in women and for liver and intestine cancers in men [13,14,15,16].

Diabetic foot is considered by several authors to be the most severe among the complications associated with diabetes [1,6]. In fact, rather than considering the diabetic foot as a single complication, it should be considered as a complex pathology in which long-term chronic complications, like neuropathy and vasculopathy, interacting with the entire organism of the patient, creates a conundrum of causative factors ending in a syndrome characterized by frailty and comorbidity [17]. Patients with diabetic foot syndrome have 2.5 times higher mortality than diabetic patients without, 5% of patients with DFS die within 1 year of their first foot ulcer and 42.2% within 5 years. Furthermore, mortality increases according to age, sex, the presence of peripheral vascular disease and chronic kidney disease [14]. The most frequent causes of death include chronic kidney disease (24.6%), cardiovascular events (19.6%) and infections (15.6%) and malignancy (9.6%) [18].

It has been also suggested that cardiovascular disease and cancer share mutual risk factors, such as obesity, diabetes mellitus, alcoholism and tobacco, which may explain, at least in part, concurrent manifestations. In addition, numerous ancillary mechanisms and pathways associated with cardiovascular disease have been shown to be involved in cancer pathogenesis and progression, like atherosclerosis, hypoxia CVD-related, chronic inflammation with the dysregulation of many cytokines [15]. Indeed, is important to underline that these pathological mechanisms underlie to diabetic foot syndrome pathogenesis too [19].

In particular, patients with DFS have a cardiovascular profile at greater risk than diabetic patients without DFS. On the one hand, they have a greater presence of cardiovascular risk factors (hypercholesterolemia, hypertriglyceridemia and microalbuminuria), on the other hand, they show a greater number of previous or new-onset cardiovascular events (coronary heart disease end cerebrovascular disease) [20,21].

This is the reason why DFS has been considered as a marker of CVD morbidity and mortality.

Following the same line of thought in relation to the common pathogenetic factors and chronicity profile, it seems realistic to hypothesize that DFS could be associated with an increasing cancer prevalence.

There are no data in the literature about the existence of a direct relationship between DFS and cancer prevalence, but only between DM and cancer [4,5,6,7].

In our study, for the first time, we have observed a significant higher prevalence of cancer in a DFS+ population, without a surprisingly significant difference between DFS− patients and non-diabetic controls.

These data could be explained as a reflection of the splitting DF from the general DM population, because in previous studies they were always mixed and counted together. In this way, we can suggest that the real pathogenetic link could be not between DM and cancer, as described in the literature until now, but between DFS and cancer itself.

We are fully aware of the limitations of our study, which is a single-centre, retrospective, and only observational study.

Moreover, the groups analysed were very heterogeneous from several points of view; for example, the cancer diagnosis occurred both in the previous clinical history of patients and also at the DFS diagnosis time.

Nevertheless, this is the first time that a possible direct correlation between DFS and cancer has observed, and we feel that our data could be of interest in view of promoting further investigations.

New prospective studies will be necessary to confirm and again investigate the relationship between cancer and diabetic foot syndrome, possibly in a multicentric setting.

## 7. Conclusions

A higher prevalence of cancer in DF patients than in DM patients and non-diabetic controls is reported. DFS, age and haemoglobin are independent factors associated with cancer.

If the association between DFS and cancer and its relatively higher prevalence will be confirmed, DFS should be considered an independent risk factor for neoplasms. Consequently, the DFS screening scenario will have to change, with the implementation of oncologic screening tests.

## Figures and Tables

**Table 1:** Clinical, biometric and biochemical data among the total, DFS+, DFS− and Controls (C) (* NA = not applicable).

	Whole Group	DFS+	DFS−	*p* DFS− vs. DFS+	Controls	*p* DFS+ vs. C
N°	700	222	223	--	255	--
Gender	M 479, F 221	M 166, F 56	M 141, F 82	0.008	M 172, F 83	0.084
Age	70.1 ± 13.7	69.6 ± 10.4	75.2 ± 11.7	<0.001	66.1 ± 16.0	0.005
Smoking habit	Y 19.9%, N 80.1%	Y 21.7%, N 78.3%	NA *	NA *	Y 19.1%, N 89.9%	0.567
Weight (Kg)	83.2 ± 17.8	86.2 ± 18.8	80.8 ± 16.7	0.017	NA *	NA *
BMI (Kg/m^2^)	28.6 ± 5.2	28.6 ± 5.0	28.6 ± 5.3	0.946	NA *	NA *
MACE	Y 40.7%, N 59.3%	Y 48.9%, N 52.1%	Y 59.4%, N 40.6%	0.027	Y 22.5%, N 77.5%	<0.001
AF	Y 16.4%, N 83.6%	Y 15.6%, N 84.4%	Y 26.9%, N 73.1%	0.007	Y 10.6%, N 89.4%	0.130
[Hb] (g/dL)	12.1 ± 5.4	11.6 ± 4.7	11.7 ± 2.1	0.993	13.2 ± 2.1	0.009
Creatinine (mg/dL)	1.09 (IQR 0.84–1.53, 0.08–13.7)	1.25 (IQR 0.95–1.76, 0.48–13.7)	1.23 (IQR 0.88–1.84, 0.08–8.08)	0.88	0.97 (IQR 0.79–1.23, 0.36–10.39)	<0.001
CRP (mg/dL)	5.90 (IQR 1.67–14.19, 0–104)	2.89 (IQR 0.56–10.26, 0–104)	10.25 (IQR 2.92–18.97, 0–46.8)	<0.001	6.30 (IQR 2.70–12.47, 0–41.38)	<0.001
PCT (ng/mL)	0.16 (IQR 0.07–0.71, 0.00–100)	0.16 (IQR 0.07–0.61, 0.04–4.66)	0.30 (IQR 0.11–1.43, 0.00–100)	6	0.12 (IQR 0.06–0.31, 0.00–47.16)	0.134
Neutrophils (10^3^/mcl)	6.05 (IQR 4.02–9.18, 0.00–41.00)	6.09 (IQR 4.27–9.39, 0.53–31.51)	7.58 (IQR 4.70–11.18, 0.46–41.00)	4	5.05 (IQR 3.36–7.36, 0.00–20.00)	<0.001
Lymphocytes (10^3^/mcl)	1.17 (IQR 0.77–1.76, 0.00–19.53)	1.68 (IQR 1.14–2.18, 0.17–19.53)	1.03 (IQR 0.71–1.58, 0.00–4.3)	<0.001	1.00 (IQR 0.64–1.35, 0.00–11.39)	<0.001

**Table 2:** Features of DM patients with (DFS+) and without (DFS−) diabetic foot syndrome.

	DFS+	DFS−	Significance
Number	222	223	---
Type of Diabetes (T1DM/T2DM)	5.4%/94.6%	2.4%/97.6%	---
HBA1C (mmol/mol)	60.8 ± 19	55.3 ± 16.6	*p* = 0.009
Age at Diagnosis	48.8 ± 15.6	58.2 ± 15.3	*p* = 0.017
Duration of Diabetes	19.0 (IQR 12.0–28.3, 0–80)	13.0 (IQR 5.0–20.0, 0.2–64)	*p* = 0.001
Insulin Therapy	Y 74.2%, N 25.8%	Y 45.1%, N 54.9%	----

**Table 3:** Type of cancer distribution.

Cancer Site	DFS+ Tot. *n*. = 46 (%)	DFS− Tot. *n*. = 26 (%)	Controls Tot. *n*. = 34 (%)
Bladder	4 (9)	2 (8)	2 (6)
Blood	9 (20)	2 (8)	1 (3)
Colon	8 (17)	2 (8)	2 (6)
Lung	2 (4)	2 (8)	3 (9)
Kidney	2 (4)	4 (15)	2 (6)
Mammary	2 (4)	5 (19)	8 (24)
Prostate	2 (4)	1 (4)	4 (12)
Skin	12 (26)	3 (12)	3 (9)
Uterus	2 (4)	3 (12)	1 (3)
Other	3 (7)	3 (12)	4 (12)
Unknown	0	2 (8)	4 (12)

**Table 4:** Univariate and multivariate regression for cancer diagnosis.

	Univariate Analysis			Multivariate Analysis		
Determinants	EXP(B)	CI 95%	*p* Value	EXP(B)	CI 95%	*p* Value
DFS+	1.787	1.172–2.723	0.007	1.740	1.088–2.784	0.021
MACE	1.000	0.587–1.702	0.999	--	--	--
Atrial fibrillation	1.175	0.670–2.058	0.574	--	--	--
Age	1.032	1.015–1.050	0.000	1.037	1.017–1.057	<0.0001
[HB]	0.862	0.782–0.950	0.030	0.924	0.830–1.028	0.146
Creatinine	1.031	0.890–1.194	0.685	--	--	--
Gender	1.359	0.885–2.087	0.161	--	--	--
HbA1c	0.989	0.972–1.006	0.211	--	--	--
CRP	0.997	0.975–1.019	0.773	--	--	--
PCT	1.018	0.990–1.046	0.214	--	--	--
Neutrophil	1.011	0.970–1.054	0.615	--	--	--
Lymphocite	1.054	0.909–1.222	0.487	--	--	--
Age at diagnosis	1.001	0.982–1.020	0.944	--	--	--
Years of DM	1.027	1.005–1.051	0.017	--	--	--
Insulin therapy	1.103	0.619–1.962	0.740	--	--	--

## Data Availability

Data are not available due to local restrictive rules.
